# Single‐cell RNA sequencing in pediatric research: Focusing on differentiation trajectories and immune microenvironment of neuroblastoma

**DOI:** 10.1002/pdi3.61

**Published:** 2024-05-23

**Authors:** RuiZong Wang, Shan Wang

**Affiliations:** ^1^ Department of Pediatric Surgical Oncology Children's Hospital of Chongqing Medical University Chongqing China; ^2^ National Clinical Research Center for Child Health and Disorders Ministry of Education Key Laboratory of Child Development and Disorders Chongqing Key Laboratory of Pediatrics Chongqing China

**Keywords:** differentiation trajectory, neuroblastoma, single‐cell RNA sequencing

## Abstract

Recently, single‐cell RNA sequencing (scRNA‐seq) has emerged as a novel and high‐resolution technique for identifying cell types, states, and subpopulations. This technique enables researchers to uncover cellular heterogeneity and detect rare cell populations that might be indistinguishable in bulk RNA‐seq data. The primary aim of scRNA‐seq analysis is to investigate cellular heterogeneity and distinguish distinct cell types or states. scRNA‐seq provides a detailed understanding of intercellular differences and diversity by obtaining gene expression data for each individual cell. Moreover, clustering methods in scRNA‐seq can be used to group cells bring into subpopulations based on their gene expression patterns, thereby uncovering similarities and differences that assist in identifying and defining cell types. Newly discovered cell types can be validated and named by labeling known cell marker genes. Additionally, scRNA‐seq helps in identifying genes specifically expressed at different developmental stages, in various tissue types, or under various disease states. Recently, there has been a growing trend in using single‐cell transcriptome sequencing technology for neuroblastoma (NB) research. Through conducting a comprehensive review of relevant articles published thus far, our understanding of NB has been significantly enriched from three critical perspectives: differentiation trajectory, tumor heterogeneity, and immune microenvironment. Firstly, in exploring the differentiation trajectory of NB, we have summarized the tumor's origin and subsequent directions of differentiation. By elucidating a complete tumor differentiation pathway, we can enhance our understanding of the mechanisms underlying spontaneous tumor regression. Secondly, we have summarized the heterogeneity of tumors, which encompasses different states, cell morphologies, and characteristic genes of NB identified through single‐cell sequencing technology. This consolidation of knowledge enhances our understanding of the heterogeneity of NB. Lastly, we have employed single‐cell sequencing technology to analyze the immune microenvironment, focusing on the cellular components within the tumor's surrounding environment and the diverse states of immune cells. This valuable information contributes to the advancement of NB diagnosis, treatment, and prognosis. In conclusion, the application of single‐cell sequencing technology in NB research has significantly advanced our understanding of the disease and carries great significance.

## 1 BACKGROUND

**FIGURE 1:**
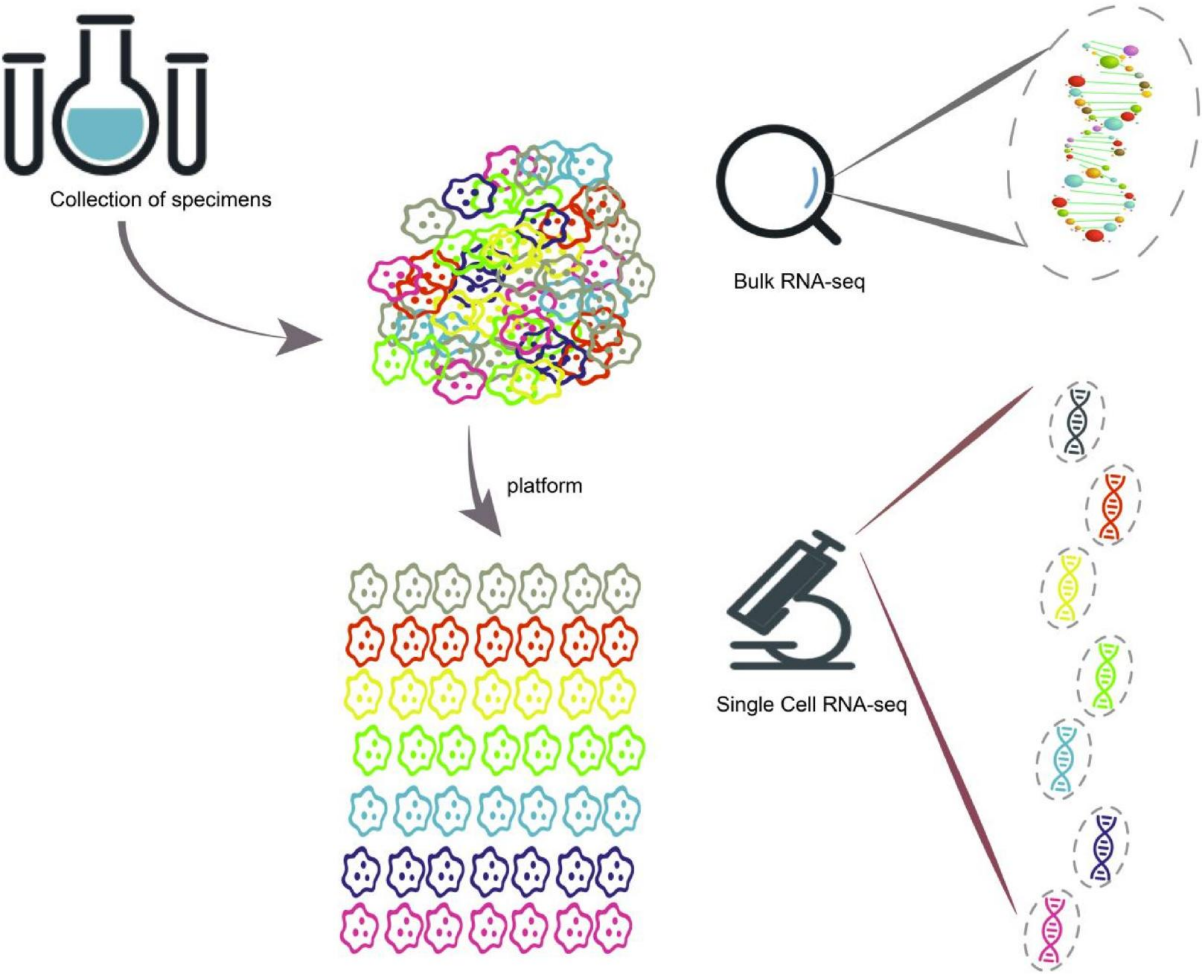
scRNA‐seq versus bulk RNA‐seq.

Traditional sequencing technology (bulk RNA‐seq) obtains information about the cell population by batch sequencing tumor cells. This reflects the overall characteristics of the cell population. However, it cannot specifically understand the heterogeneity of tumor cells. Single‐cell RNA sequencing (scRNA‐seq) technology allows for mechanistic studies at the molecular level by analyzing the gene expression profile of each individual cell. It can detect tumor cell heterogeneity, identify rare cells, divide cell subsets, track cell lineages, and reveal the differentiation trajectory (Figure 1). Furthermore, it can locate mutated genes and discover new tumor biomarkers on a single‐cell level.^1^ This technology can also be used to construct transcriptome databases of different tumor cell subsets, thereby enhancing our fundamental understanding. This can deepen our understanding of tumor biology by elucidating the origin and mechanism of tumorigenesis, exploring the trajectory and process of growth, revealing the heterogeneity of cell subsets in the tumor microenvironment (TME), and analyzing the extent of immune cell infiltration and tumor antigen expression.^2^, ^3^, ^4^, ^5^, ^6^, ^7^, ^8^ In summary, the primary distinction between bulk RNA‐seq and scRNA‐seq lies in the scale and resolution of the analysis. Bulk RNA‐seq provides an average expression signature of a cell population, whereas scRNA‐seq enables the study of gene expression at the individual cell level, unveiling cellular heterogeneity and identifying rare cell types or states. Mainstream single‐cell sequencing platforms include 10× Genomics Chromium, BD Rhapsody, DNBelab C4, and Singleron Matrix.

Neuroblastoma (NB), the most common extracranial solid tumor in children, is a heterogeneous tumor originating from the sympathetic nervous system. NB tumors typically occur in the abdomen, specifically in the adrenal glands. Some studies have shown that NB is a tumor caused by errors in the evolution of neural crest cells (NCCs) during fetal development, making it also considered an embryonic tumor.^9^

In the United States alone, approximately 700 cases are diagnosed each year, with the majority occurring in children under 5 years of age. Specifically, the median age of diagnosis for these children is only 17 months.^10^

The incidence of NB is 1.2 cases per 100,000 people, and due to the high incidence of drug resistance and relapse among patients, the survival rate of NB is less than 50%, with an associated mortality rate of about 15% among all childhood cancer deaths.^11^, ^12^, ^13^, ^14^, ^15^, ^16^, ^17^, ^18^ As a heterogeneous disease, NB demonstrates varying prognostic survival rates in patients of differing risk levels. Low‐risk patients (LR) have a survival rate exceeding 90% with a positive prognosis. However, high‐risk (HR) patients still have a 5‐year survival rate below 50%.^19^, ^20^

NB represents childhood embryonal tumors with distinct clinical features, as evidenced by the fact that only a small proportion can resolve spontaneously. Highly malignant NB can invade peripheral blood vessels and organs during the early stages of the disease process, and can even metastasize to the bone, bone marrow, and lymph nodes.^21^ Although key genetic factors for the pathogenesis and progression of NB have been identified, such as amplification of MYCN, deletion of TP53, mutation or amplification of ALK, rearrangement of TERT, deletion or mutation of ATRX, and segmental chromosomal aberrations,^22^ it remains challenging to provide a clear and systematic explanation of NB's pathogenesis.

With the continuous advancement of technology, single‐cell sequencing technology can help us gain a deeper understanding of NB. This review provides a concise summary of significant scRNA‐seq studies^23^, ^24^, ^25^, ^26^, ^27^, ^28^, ^29^, ^30^, ^31^ conducted in NB and explores how these studies have contributed to our understanding of the molecular mechanisms underlying this disease. The profiling has significantly contributed to our understanding of the molecular underpinnings of NB. We will provide a systematic elaboration on differentiation trajectory, heterogeneity, and immune microenvironment of tumor cells in NB.

## 2 DIFFERENTIATION TRAJECTORIES OF NB TUMOR CELLS

Since NB is widely distributed, with adrenal NB accounting for approximately 47% of cases, this study focuses on adrenal NB.^32^ Consequently, the localization of the tumor in the adrenal region suggests a correlation between adrenal development and tumor cell formation. NB tumors are considered to be a result of deviations during the development of the adrenal medulla in the embryonic period. The displacement of sympathetic blasts from the embryonic neural crest (ectoderm) can lead to the formation of the adrenal medulla. Therefore, trunk NCCs present on the dorsal side of the aorta are considered to be sympathetic adrenal progenitor cells, as well as the source of adrenal medulla development.^33^ During its development, the NB tumor differentiates into two types of cells: (1) sympathetic blasts, which mature into sympathetic ganglion cells, and (2) chromoplasts, which mature into chromaffin cells. The collective term for these cells is NBs (Figure 1). It is currently understood that NB is a result of migration and differentiation of NCCs into sympathetic neurons. Furthermore, the presence of adrenergic components in NB, similar to healthy sympathoblasts, suggests that immature sympathetic nerve cells or their progenitor cells can also give rise to tumors.^34^

Initially, research focused on Schwann cell precursors (SCPs) and recent cell lineage tracing studies indicate that SCPs produce chromaffin cells at a relatively late stage, coinciding with the formation of NB tumors.^35^ Single‐cell sequencing of embryonic and fetal adrenal glands revealed different morphologies, including early NCCs and related derivatives, late neural crest and SCP‐derived cell types, chromaffin cells, and adrenal sympathetic blasts. Further analysis showed that differentiated chromaffin cells and sympathetic nerve cells were in a terminal state without further differentiation potential.^23^ Of all the progenitor cells found in the human fetal adrenal medulla, SCPs evolved from NCCs were identified as the common progenitors of chromaffin and sympathetic blast progenitors, suggesting a connection between the production of NB tumor cells and SCP differentiation.^30^, ^36^ SCPs act as transitional cells during the differentiation process from NCCs to the adrenal medulla, eventually evolving into chromaffin cells after an intermediate proliferation stage.^23^ This finding supports the existence of a "bridge" cell state through which SCPs transition into chromaffin cells.^37^ In summary, adrenal medullary cells are produced through the evolution of SCPs, ultimately giving rise to both nerve cells and chromaffin cells. This connection between progenitor cells and NBs can be observed in different subtype tumors.^29^, ^30^

However, a recent study presented different findings, suggesting that SCPs are not the common progenitor cells of chromaffin cells and sympathetic nerve cells. In mice, chromaffin cells and sympathetic neurons have different origins, with chromaffin cells being produced by SCPs and sympathetic neurons evolving from the original NCC cells.^38^ Although SCPs undergo differentiation toward chromaffin cells in most cases, they rarely differentiate into sympathetic nerve cells.^36^ Furthermore, the postnatal multiplication and development pattern of chromaffin cells in the adrenal glands remains unknown. A study on prenatal development in mice concluded that both SCPs and NCCs can produce chromaffin cells and sympathetic nerve cells.^39^

By analyzing single‐cell sequencing results of primary adrenal NB, it was discovered that tumor cells predominantly exhibit a chromaffin cell‐like phenotype, indicating that neuroendocrine cells act as malignant cells in primary NB tumors.^23^ These findings are consistent with other analyses that detected only cancerous adrenal medullary cells in the NB tumor group, with no cancerous changes observed in other cell types. These results support the claim that adrenal medullary‐like cells represent malignant cells in primary NB tumors.^24^

However, these findings contradict a recent study that suggests neuroblasts are the most susceptible to cancer among all cell types in the adrenal gland. This study proposed that NB originates from NCC cells and found that tumor cells exhibit a transcriptional state similar to that of sympathetic nerve cells during malignant transformation.^40^ The contradiction may arise from differences in cell type annotations of normal adrenal medullary cells. Based on a combination of experimental and single cell transcriptomics‐based evidence, another study demonstrated that SCPs in mouse embryos differentiate into immature chromaffin cells, which then evolve into sympathetic neurons. In humans, SCPs can transform directly into chromaffin cells and sympathetic neurons. Additionally, the results indicate that these sympathetic nerve cells can transform into chromaffin cells. Remarkably, during the development of the sympathetic adrenal system, SCPs can produce not only sympathetic neuroblasts but also chromaffin cells derived from immature sympathetic nerve cells.^31^ These findings reflect a dynamic change process, which may explain the occasional detection of oncogenic transformation in neuroblasts. In conclusion, the chromaffinocyte‐like phenotype of tumor cells in NB is supported, as they directly derive from sympathoblasts. Additionally, the study by Liu et al. found that malignant tumor cells can differentiate into fibroblasts (Fib).^24^

**FIGURE 2:**
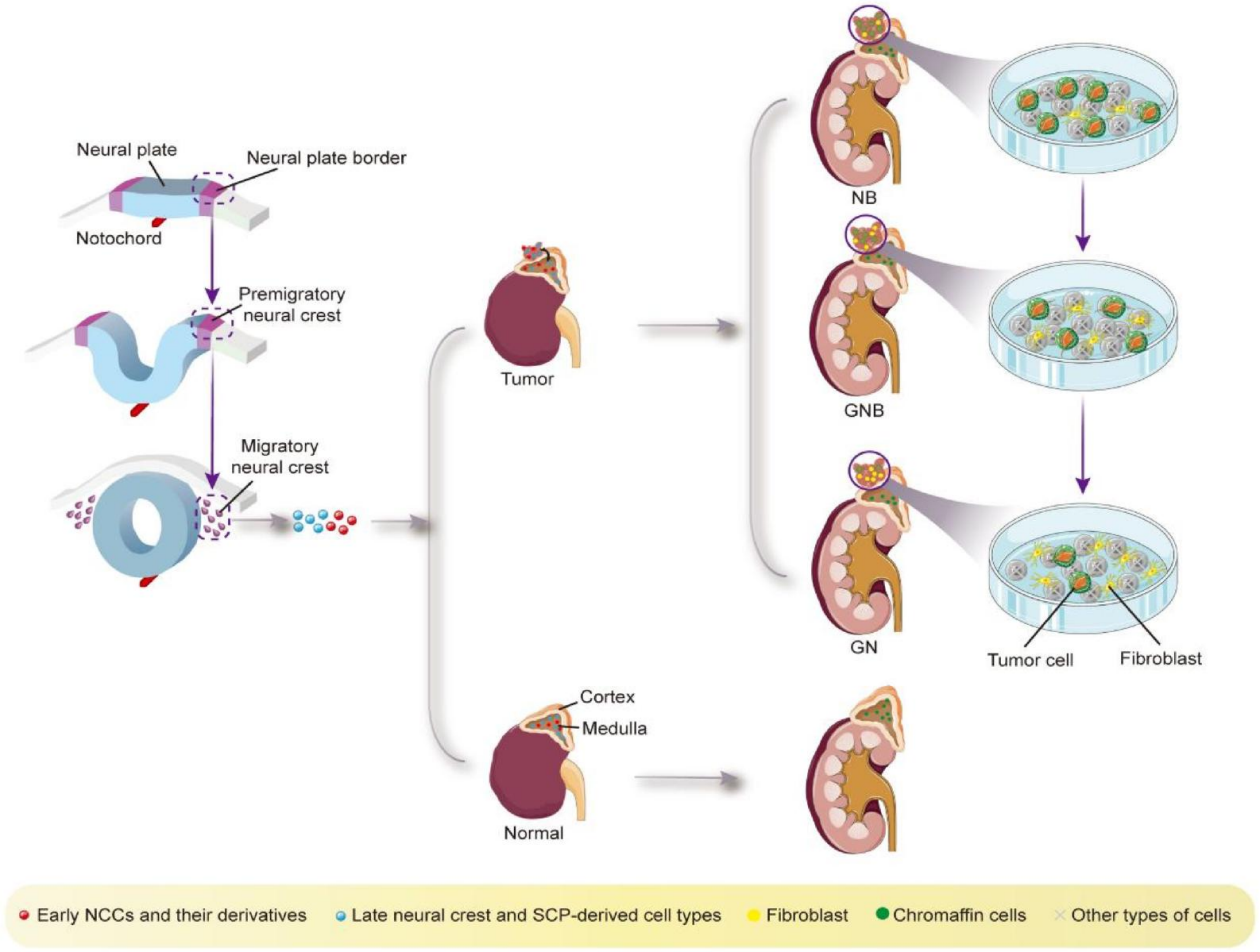
In the process from NB to GNB to GN, tumor cells (NE cells with chromaffinoid phenotype) gradually decrease, and fibroblasts gradually increase.

The International Neuroblastoma Pathology Commission categorizes NBs into four broad types: NBNB, ganglionic NB (GNB) divided into nodular and mixed subtypes, ganglioneuroma (GN), which represents a mature differentiation stage, and ganglioneuroblastoma (GNB) that serves as a transitional stage between neuroblastoma and ganglioneuroma. NB is the most primitive and undifferentiated subtype, while ganglioneuroma is fully mature. The transitional stage between these two is represented by ganglion NB. Within the primitive category, there are subtypes based on the degree of differentiation of the mother cells, including undifferentiated, poorly differentiated, and differentiated types. In summary, the differentiation trajectory of NB begins with NCCs, then progresses through SCPs, tumor cell NEs, and eventually differentiates into Fibs. This understanding contributes to our knowledge of differentiation therapy for NB and the process of spontaneous regression (Figure 2).

## 3 HETEROGENEITY OF NB TUMOR CELLS

NB is characterized by heterogeneous cell populations consisting of tumor cells, stromal cells, and infiltrating immune cells with diverse genotypes and phenotypes. This cellular heterogeneity contributes to variations in tumor proliferation rates, invasion capacities, and drug sensitivities, all of which have significant implications for the diagnosis, treatment, and prognosis of patients. Previous studies have highlighted the presence of temporal heterogeneity in NB cells, characterized by differential molecular biomarkers at the time of diagnosis and recurrence, as well as spatial heterogeneity within the TME. Further investigation can reveal potential therapeutic targets and facilitate risk stratification and precise treatment of NB. Avitabile et al. demonstrated that distinct NB subpopulations exhibit unique gene expression profiles, allowing for a better understanding of drug resistance and heterogeneity through single‐cell sequencing analyses.^26^ Subsequently, we will investigate NB heterogeneity in terms of cell morphology and gene expression states.

### 3.1 Differentiation heterogeneity following NB tumor formation

NB cells have been found to exhibit two distinct epigenetic states that can be identified based on the presence of super‐enhancer elements.^27^, ^41^, ^42^ Specifically, one population consists of mesenchymal or NCCs with a low degree of differentiation, while the other population consists of adrenergic and sympathetic norepinephrine cells with a higher degree of differentiation. These two states, referred to as MES and ADRN, respectively, coexist in human NB and can undergo spontaneous transformation between each other.

Malignant tumor cells are associated with a specific phase during the transition between adrenergic and mesenchymal states, and recent studies have identified an intermediate state characterized by an aggressive neurodevelopmental phenotype. This intermediate state is strongly linked to increased tumor cell proliferation, dissemination, drug resistance, and recurrence risk.^43^

Moreover, investigations using human and mouse tumor models have revealed a substantial presence of the MES state in NB cases with metastasis and recurrence. Furthermore, tumors in the MES state exhibit heightened resistance to chemical treatments in vitro, suggesting that under drug stimulation, intermediate state cells are inclined to differentiate into the MES state as a means of evading drug cytotoxicity. Consequently, precise targeting of MES subsets represents a crucial strategy for tumor therapy and recurrence prevention.^25^, ^43^ Furthermore, this study observed that human NB cells with a greater abundance of MES state characteristics exhibit higher levels of basal inflammatory transcripts compared to cells with differing state proportions. This suggests that cells in the MES state may display heightened sensitivity and vulnerability to the effects of inflammatory stimuli, rendering them more responsive to basic immunotherapy interventions. Additionally, the study identified the integral role of T cells in the upregulation of basal inflammatory signaling and the perception of MES status.^25^ Conversely, Boeva et al. demonstrated that cellular properties in the ADRN state are determined by core regulatory circuits (CRCs), involving transcription factors such as PHOX2B, HAND2, and GATA3.^27^ Dong et al. discovered a significant correlation between tumor differentiation grade and the degree of differentiation in the chromaffin cell subpopulation. The study showed that the higher the differentiation degree, the better the prognosis for the patient. Therefore, the degree of differentiation of the chromaffin cell subset in the ADRN state is correlated with NB heterogeneity.^23^

### 3.2 Differentiation heterogeneity prior to NB tumor formation

Epithelial‐mesenchymal transition (EMT) occurs during early vertebrate embryo development, specifically generating NCC precursors from the dorsal part of the neural tube (NT) that undergo EMT.

Dong et al. observed a strong correlation between MYCN amplification in tumors and EMT NCC status. Subsequent studies also found associations between MYCN overexpression and upregulation of the TWIST1 gene.^23^ TWIST1, which regulates EMT and serves as a marker gene for EMT NCC, was found to be upregulated in MYCN‐amplified tumors.^44^ Recent research has shown that TWIST1 and MYCN cooperate to enhance oncogene transcription,^45^ supporting the association between MYCN and EMT NCC‐related genes in MYCN‐amplified tumors.^23^ MYCN's role in tumor development differs in low‐risk or highly differentiated tumors compared to HR (MYCN‐amplified) tumors where MYCN induces dedifferentiation and proliferative activation.^30^ MYCN heterogeneity is also observed among different cell lines, such as BE2C and Kelly cells, which are commonly used models for MYCN amplification. These cell lines exhibit mesenchymal properties and upregulation of the TWIST1 and DNAJC1 genes, indicating heterogeneity between different cell lines. Overall, these findings emphasize the importance of single‐cell sequencing for exploring NB heterogeneity.^28^

Currently, research on NB heterogeneity conducted by SCPs and UCHCs is primarily descriptive. For SCPs, their focus has mainly been on understanding the relationship between SCPs and NB staging and prognosis. It has been observed that higher numbers of SCPs are associated with improved survival in INSS stages 1, 2, and 3. However, the relationship between SCP characteristics and survival is not significant in stages 4 and 4S. This suggests that healthy SCPs in early‐stage tumors may have a protective effect.^29^ Speaking of UCHCs, we refer to circulating chromaffin cells as undifferentiated chromaffin cells (UCHCs) and non‐circulating chromaffin cells as differentiated chromaffin cells. The study revealed a significant association between a higher proportion of mature neuroblast‐like tumor cells and favorable clinical outcomes. Conversely, a high proportion of UCHCs was associated with a poor prognosis. These findings confirm the correlation between a high proportion of mature neuroblasts and a low proportion of UCHCs with positive clinical outcomes. Both markers can independently provide prognostic information about NB patients.^30^

### 3.3 Heterogeneity of NB genes

Genomic studies of NB have identified several characteristic genes that contribute to its heterogeneity. The differential gene SIX3, discovered through genetic comparison between NB cells and normal neuroblasts, functions as a regulatory gene during embryonic development of the forebrain and eyes. Due to the limited expression of certain genes in NB only during embryonic development, SIX3 presents as a potential therapeutic target.^46^ Another transcription factor, TFAP2B, is highly expressed in normal neuroblasts but not in HR NBs. It restores the differentiation profile of poorly differentiated tumors.^47^ In low‐risk tumors, preserved TFAP2B allows for normal differentiation processes but inhibits these processes.^30^ Therefore, TFAP2B may serve as a prognostic molecule. NTRK1 expression is considered a positive clinical prognostic indicator and a low‐risk marker, while NTRK2 results show the opposite.^36^, ^48^ Additional observational studies have found that low‐risk tumors are more common in children under 18 months old.^49^ This suggests a potential correlation between the tumor and the nature of the progenitor cell population derived at this age, leading to the clinical use of age as a prognosis indicator.^36^ In‐depth research and gene comparison on tumor cells conducted by Liu et al. identified TUBA1A and STMN2 as highly expressed prognostic markers associated with poor outcomes in the NB group. On the other hand, ITGB1 in M2‐like macrophages is associated with tumor cell genesis and is linked to good prognosis, making it a potential prognostic stratification marker and therapeutic target.^24^

## 4 TUMOR IMMUNE MICROENVIRONMENT IN NB

The TME encompasses various components, such as blood vessels, immune cells, fibroblasts, signaling molecules, and extracellular matrix. Tumors closely interact with and are influenced by the surrounding microenvironment. The TME is a complex environment where cancer cells interact with immune cells and stromal cells through numerous biochemical and physical signals, which play a crucial role in cancer progression and metastasis. Immune cells and mesenchymal cells are vital components of the TME, and they significantly impact NB survival, proliferation, and therapeutic resistance.^50^, ^51^, ^52^, ^53^ Hence, determining the specific TME components in NB is crucial for enhancing current and future immuno‐oncology therapies for pediatric patients. Analysis of single‐cell sequencing results reveals that the predominant immune cells in the TME are T cells and bone marrow cells. Notably, the proportions of immune cell types vary among patients, indicating a possible correlation between the differential expression of immune cell genes and survival rates.^54^ Yuan et al. discovered that NB exhibits a lower relative abundance of tumor‐infiltrating lymphocytes and a higher abundance of noninflammatory macrophages. This finding suggests a potential association between immune evasion, tumorigenic microenvironment, and aggressive behavior of NB.^43^

### 4.1 Role of myeloid cells in the NB immune microenvironment

Myeloid cells, which frequently invade tumors and promote tumor growth, have been linked to poor clinical prognosis in NB.^55^, ^56^, ^57^ Costa et al. identified immunosuppressive myeloid TME in TH‐MYCN mouse tumors. Interestingly, myeloid cells were associated with improved survival.^58^, ^59^ Further analysis uncovered the predominant subtype of myeloid cells as macrophages, which display heterogeneity across various tumor types.^58^, ^60^ Monocytes and macrophages can express different subtypes of myeloid cells, including a pro‐inflammatory phenotype in some cases.^59^, ^61^ Pro‐inflammatory immune cells and tumor inflammation can facilitate tumor growth, metastasis, and spread.^62^ However, Verhoeven et al. argue that a pro‐inflammatory status can serve as a favorable prognostic factor, being positively associated with survival.^54^

### 4.2 Role of T cells in the NB immune microenvironment

In the TH‐MYCN NB model, immunosuppressive myeloid suppressor cells (MDSCs) display a myeloid immunosuppressive effect, inhibiting T cell proliferation and causing malignant depletion of T lymphocytes in vitro. Similarly, immunosuppressive functions have been observed in human NB tumors, where macrophages are also involved.^58^ The immune microenvironment in the MYCN‐amplified NB mouse model contained a small number of T cells, macrophages with diverse phenotypes, and cell populations with MDSC phenotypes. Notably, the molecular characteristics of these populations differed from those of various macrophage subpopulations. This suggests a significant overlap and specificity between mouse and human MDSC populations, elucidating the heterogeneity and complexity of the NB macrophage subpopulation.^60^, ^63^ Subsequent studies have demonstrated an elevation in the heterogeneity of receptor proteins on T cells in the microenvironment of mouse tumors. Conversely, the microenvironment of human tumors exhibits malignant depletion of T cells associated with increased expression of inhibitory receptors and decreased expression of effector cytokines, providing functional evidence of the immunosuppressive properties of MDSCs in NB and their detrimental effects on T lymphocytes.^58^ Extensive investigation of T cells revealed distinct T cell subtypes associated with different NB prognoses. These subtypes include CTL‐1, CTL‐3, CTL‐4, naïve T cells, and Th17 subtypes.^54^, ^64^

Surprisingly, regulatory T cells (Tregs) showed no association with survival, which differs from the Treg inhibition and effector cell depletion observed in adult cancers.^65^ Additionally, our findings indicate that dendritic cells (DCs) correlate with T lymphocyte infiltration at both the transcriptional and translational levels and this correlation is related to a favorable prognosis.^24^, ^66^

### 4.3 Receptor‐ligand interaction in the NB immune microenvironment

Analysis of receptor‐ligand interactions between tumor/stroma and immune cells revealed an association between SEMA6D‐TREM2 expression and poor prognosis, highlighting a novel potential therapeutic target.^54^ CD24 expression has been observed in various solid tumors due to its inhibitory effect on phagocytosis in ovarian and breast cancers. Interestingly, CD24 surface receptors are exclusively expressed in tumor cells and can interact with SIGLEC10 on immune cell macrophages. Based on these findings, we propose that CD24/SIGLEC10 could serve as a novel target for cancer immunotherapy, potentially enabling the investigation of monoclonal anti‐CD24 antibodies as a new treatment for NB.^67^

## 5 LIMITATIONS OF THIS REVIEW

The models utilized for single‐cell sequencing technology were derived from different species, lacking uniformity. Furthermore, this review provides only a basic summary of the prenatal differentiation trajectory, with a notable absence of observation and research on the postnatal differentiation trajectory. Additionally, the summary of heterogeneity and the immune microenvironment is insufficiently comprehensive, limited to a summary of the article's conclusions, and lacks innovative insights.

## AUTHOR CONTRIBUTIONS

RruiZong Wang and Shan Wang was responsible for the overall conception and design of the article. RruiZong Wang performed data analysis, collected the data, and wrote the manuscript. Shan Wang revising the manuscript.

## CONFLICT OF INTEREST STATEMENT

The authors declare no conflict of interest.

## ETHICS STATEMENT

All articles and data used in the study have been authorized by the ethics committee of the respective institutional review committee with written informed consent from all participants in a separate study. Therefore, no additional ethical approval or informed consent is required.

## Data Availability

The data that support the findings of this study are available from the corresponding author upon reasonable request.
